# Monthly tegafur–uracil maintenance for increasing relapse-free survival in ypStage III rectal cancer patients after preoperative radiotherapy, radical resection, and 12 postoperative chemotherapy cycles: a retrospective study

**DOI:** 10.1186/s12885-019-6019-0

**Published:** 2019-08-17

**Authors:** Yi-Hung Kuo, Chia-Hsuan Lai, Cheng-Yi Huang, Chih-Jung Chen, Yun-Ching Huang, Wen-Shih Huang, Chih-Chien Chin

**Affiliations:** 1 Division of Colon and Rectal Surgery, Department of Surgery, Chang Gung Medical Foundation, Chiayi Branch, No. 6, Sec. West, Chia–Pu Road, Putz City, Chiayi Hsien 613, Chiayi, Taiwan; 2 Department of Radiation Oncology, Chang Gung Medical Foundation, Chiayi Branch, Chiayi, Taiwan; 3 Graduate Institute of Clinical Medicine, Chang Gung University, Linkuo, Taiwan

**Keywords:** Tegafur–uracil, Maintenance, Relapse-free survival, ypStage III rectal cancer

## Abstract

**Background:**

Current advancements in neoadjuvant therapy and total mesorectal excision have engendered increased local control. However, the survival benefit of preoperative radiotherapy (RT; 5 × 5 Gy) in rectal cancer patients remains inadequate, primarily because of systemic recurrence. In this retrospective single-center study, the effects of monthly tegafur–uracil maintenance (≥6 cycles) after 12 fluorouracil-based adjuvant chemotherapy cycles on 3-year relapse-free survival (RFS) was estimated in ypStage III rectal cancer patients.

**Methods:**

Of ypStage III rectal cancer patients who received preoperative RT (5 × 5 Gy) in January 2006–December 2015, those who had ypStage III cancer after preoperative radiation, radical resection, and postoperative chemotherapy were enrolled; excluded patients had ypStage I and II rectal cancer, had double cancer, had synchronous distant metastasis, had local excision, received preoperative chemoradiation, and were lost to follow-up within 1 year after cancer treatment. Included patients received either maintenance therapy or observation after postoperative chemotherapy. The primary endpoint was the effect of maintenance therapy on 3-year RFS. We set the median follow-up duration to be 69.7 (range, 15.4–148.3) months.

**Results:**

Of 259 ypStage III rectal cancer patients, 102 (59 men and 43 women) were enrolled based on the inclusion criteria. The maintenance and observation groups comprised 55 and 57 patients, respectively (mean age = 62.2 and 65.7 years, respectively; *p* = 0.185). The 3-year RFS observed in the maintenance group (85.1%) was longer than that observed in the observation group (67.5%; *p* = 0.039). Multivariate analysis proved the following to be independent prognostic factors for RFS: higher metastatic lymph node ratio (LNR ≥0.3), tegafur–uracil maintenance (≥6 cycles), and lower rectal cancer (< 6 cm from the anal verge). The higher the rectal cancer location (≥6 cm from the anal verge) was, the higher the tegafur–uracil maintenance survival benefit became (*p* = 0.041). Moreover, lower cancer location (< 6 cm from the anal verge) and LNR ≥0.3 were both associated with a trend of longer RFS after tegafur–uracil maintenance therapy (*p* = 0.164 and 0.113, respectively).

**Conclusions:**

After the execution of fluorouracil-based adjuvant chemotherapy, administering monthly tegafur–uracil (≥6 cycles) may improve the 3-year RFS of ypStage III rectal cancer patients.

## Background

The incidence of colorectal cancer—which, in Taiwan, is the third-leading cause of deaths related to cancer—has risen over the past decades from 38 to 70 and 30 to 51 per 100,000 men and women, respectively [1]. The surgical treatment of middle and lower rectal cancer is more technically challenging compared with that of colon cancer because the pelvic cavity is narrow. In addition to the technical challenge, the prognosis of locally advanced colorectal cancer is poorer than that of locoregional lymph node metastasis [2]. A preoperative imaging study [3] reported that preoperative short-course radiotherapy (RT; radiation dose: 2500 cGy) or chemoradiotherapy (CRT; radiation dose: 5040 cGy) presently constitutes the benchmark for the treatment of locally advanced rectal cancers, including clinical T3, T4, or positive-node stage metastasis as determined in accordance with the tumor–node–metastasis (TNM) classification system. However, in rectal cancer treatment, compared with preoperative long-course CRT or short-course RT, the effectiveness of adjuvant chemotherapy remains unclear. Over the past two decades, adjuvant chemotherapy comprising either 5-fluorouracil plus leucovorin alone (5FU/LV) or combined with oxaliplatin (FOLFOX) has been strongly recommended for treating stage III colon cancer [4, 5]. Recently, controversy remains concerning the effect exerted by adjuvant treatment with total mesorectal excision (TME) on overall survival (OS) or disease-free survival (DFS) in rectal cancer at ypStage II and III [6–9]. A review article reported that relevant trials have not reported favorable results for adjuvant chemotherapy performed postoperatively in rectal cancer patients after preoperative chemoradiation [8]. In different age groups (< 60 vs. ≥60 years), the addition of oxaliplatin demonstrated varied effects on the DFS and OS of rectal cancerpatients [9]. The National Comprehensive Cancer Network guideline recommends that after preoperative long-course CRT or short-course RT and curative surgery for ypStage III rectal cancer, adjuvant chemotherapy should be administered. However, adjuvant therapy regimens are majorly based on studies on cohorts involving patients diagnosed as having stage III colon or colorectal cancer [10, 11].

The dihydropyrimidine dehydrogenase inhibitor tegafur–uracil (TTY Biopharm Co, Taiwan), which is administered orally, provides a survival benefit similar to that of 5FU/LV infusion, based on the DFS and OS of patients who were diagnosed as having stage II and III colon cancer [12]. Previously executed research has reported equal toxicity and good compliance between the two regimens. In addition, the use of oral anticancer drugs is associated with advantages, including continuous drug release and feasible patient tolerance, which can exert an efficient effect on cancer treatment.

In the neoadjuvant treatment and TME era, positive OS benefit was demonstrated by the Swedish Rectal Cancer Trial alone; despite increased local control, neoadjuvant RT has not shown a survival benefit because systemic recurrence remains a significant problem in rectal cancer [13, 14]. Our hypothesis was that in ypStage III rectal cancer patients, the survival is improved by adjuvant chemotherapy or further maintenance therapy. However, evidence of the survival benefit of monthly tegafur–uracil maintenance after adjuvant chemotherapy in colorectal cancer patients is unavailable. This paper presents the initial results of a retrospective assessment of the 3-year relapse-free survival (RFS) and 5-year cancer-specific survival (CSS) as well as efficacy data associated with tegafur–uracil maintenance. Moreover, we evaluated the survival benefit in patients diagnosed as having ypStage III rectal cancer who, after adjuvant chemotherapy, received tegafur–uracil maintenance or observation.

## Methods

### Patient selection

We applied a retrospective enrollment process, thus enrolling 259 patients who were diagnosed as having locally advanced rectal cancer, which was defined as clinical T3 or T4 or as a node-positive condition in accordance with the TNM system. The modality of preoperative imaging for every rectal cancer patient included nuclear magnetic resonance imaging and abdomen and chest computed tomography. Over January 2006–December 2015, all the included patients with rectal cancers of the lower or middle rectum received preoperative RT (5 × 5 Gy) at Chang Gung Medical Foundation, Chiayi Branch. The data were provided by the cancer registry of our cancer center. Cancers were staged using the American Joint Committee on Cancer (AJCC) Cancer Staging Manual (Seventh Edition).

### Inclusion criteria

Patients were included if they

1. had pathologically confirmed adenocarcinoma in the middle and lower rectum,
2. had ypStage III cancer as determined in accordance with the TNM system (i.e., T_1–4_, N-positive, and M0; because the collected data were based on the sixth edition as well as the seventh edition of the AJCC Cancer Staging Manual, our pathology data were restaged as defined in its seventh edition),
3. had no disease recurrence as determined by imaging and tumor marker assessments after they completed of 12 adjuvant chemotherapy cycles,
4. received ≥6 cycles of monthly tegafur–uracil maintenance, and
5. had an Eastern Cooperative Oncology Group (ECOG) status in the range of 0–2.

### Exclusion criteria

Patients were excluded if they

1. had possible synchronous distant metastasis on rectal cancer diagnosis (*n* = 34),
2. had double cancer at the first diagnosis of rectal cancer or double cancer in their medical history (*n* = 4),
3. received nonradical resection (e.g., local excision or R1/R2 resection) after preoperative RT (*n* = 8),
4. were lost to follow-up within 1 year after cancer treatment (n = 19), or
5. received preoperative CRT (*n* = 2).

Of the 192 remaining eligible patients, 33, 57, and 102 had ypStage I, II, and III cancer of the rectum, respectively. Finally, a total of 102 patients with ypStageIII cancer of the rectum after preoperative RT, curative surgery, and adjuvant chemotherapy were enrolled to analyze the effect of monthly tegafur–uracil maintenance therapy on their 3-year RFS and 5-year CSS. These patients’ data were followed until September 2018.

### Preoperative RT, radical resection for rectal cancer, and tegafur–uracil therapy

Preoperative RT comprised a 5 × 5-Gy radiation dose delivered in five fractions. A total of 102 enrolled patients underwent elective radical resection after RT. The median time interval between RT and radical resection was 6 (range, 1–24) days. The definitions of involved distal and circumferential margins were as follows:

1. Involved margin: existence of a tumor within ≤1 mm of the circumferential margin [15].
2. Involved distal margin: existence of a tumor in the resection line or positive doughnut of anastomosis [16].

All enrolled patients had curative and R0 resections (i.e., clear distal and circumferential margins).

The 5FU-based adjuvant chemotherapy was administered 4–6 weeks after curative cancer surgery. On the first day (also designated as day1), a 5-FU bolus infusion (400 mg/m^2^), followed by continuous infusion (2400 mg/m^2^) performed over a period of 46–48 h, was administered. The clinical physicians repeated every cycle every fortnight. After the execution of 12 cycles of adjuvant treatment, the clinical physicians prescribed tegafur–uracil maintenance according to patients’ performance, pathologic risk factors, and age because no evidence exists for maintenance tegafur–uracil administration in patients diagnosed as having ypStage III cancer of the rectum. Patients undergoing maintenance therapy received tegafur–uracil and folinic acid monthly. The total daily dose of tegafur–uracil (100 mg/capsule) was 400 mg divided into two doses, administered over days 1–28, every 5 weeks. Folinic acid (15 mg/tablet) was administered at 30 mg daily divided into two doses for days 1–28, every 5 weeks. There is no consensus on the use and duration of tegafur–uracil maintenance because of the absence of clinical studies. In our study, some physicians prescribed maintenance of tegafur–uracil for ≥6 months (median, 10 [range, 6–19] months) after adjuvant chemotherapy and some physicians considered observation after adjuvant chemotherapy.

### Endpoint and follow-up

After treatment completion, physical examination and carcinoembryonic antigen level testing were executed in all patients every 3 to 4 months during the first 3 years and subsequently every 6 months since year 4. Colonoscopy was performed once in the first year and thereafter every 2 to 3 years. Abdomen and chest computed tomography was performed annually in the first 3 years after rectal cancer treatment and then was performed based on clinical evaluation and tumor marker follow-up.

In this study, we defined RFS to be the symptom-free period after the end of radical surgical treatment for a cancer. Our primary endpoints were the 3-year RFS and the independent factors for RFS. The second endpoint was the 5-year CSS, the definition of which was the time spanning from the diagnosis date to the date of cancer-related death or the last day the patient was identified as being alive.

### Statistical analysis

To perform a comparison of quantitative data, we executed Fisher’s exact test as well as Pearsonchi-square test; moreover, we applied the Kaplan–Meier approach to derive CSS and RFS. We additionally plotted Kaplan–Meier survival curves for our various groups and compared them by executing log-rank tests. The confounders were controlled for by using a Cox regression model in multivariate analysis. All relevant two-tailed *p* values were calculated, with *p* < 0.05 being deemed in this study as statistically significant.

## Results

### Characteristics of enrolled patients

In this series, the 102 enrolled patients who received a diagnosisof ypStage III rectal cancer and were subjected to preoperative RT, radical resection, or TME as well as complete 5FU-based adjuvant chemotherapy were stratified into two groups: (1) the first group (also denoted as maintenance group) comprised 55 (53.9%) who patients received further maintenance therapy with monthly tegafur–uracil for ≥6 cycles; (2) the second group (also denoted as observation group) comprised the remaining 47 (46.1%) patients who received observation after 5FU-based adjuvant chemotherapy. The enrolled patients’ surgical complications were classified according to the Clavien-Dindo Classification (https://www.assessurgery.com/clavien-dindo-classification/). The treatment-related side effects from tegafur–uracil were adequately tolerated; there was no side effect severer than grade 4 toxicity. Four patients in the observation group used tegafur–uracil for < 6 cycles due to allergy and severe diarrhea.

In general, the human rectum is divided into the upper, middle, and lower rectum, located at 0–6, 7–11, and 12–15 cm from the anal verge, respectively [17]. Our database defines the tumor location based on preoperative colonoscopy. The characteristics of the patients in the two groups are listed in Table 1. Both patient groups were determined to comprise 43 women and 59 men; intergroup differences in sex distribution were determined to be nonsignificant (*p* = 0.743). The mean ages were 62.2and 65.7 years in the maintenance and observation groups, respectively (*p* = 0.185). Except for the examined lymph node number (ELN), the other continuous variables of the maintenance and observation groups did not differ significantly (Table 2).

**Table 1:** Clinicopathological characteristics of patients with or without UFUR maintenance after adjuvant chemotherapy for ypstage III rectal cancer

Variable Category	Patient number (%) of each category in variable	Patients with UFUR maintenance	
		Patient number (%) in each category	*P value*
Age group			
< 65 years	45 (44.1)	26 (57.8)	0.488
≥ 65 years	57 (55.9)	29 (50.9)	
Sex			
Female	43 (42.2)	24 (55.8)	0.743
Male	59 (57.8)	31 (52.5)	
Tumor location			
< 6 cm	46 (45.1)	27 (58.7)	0.381
≥ 6 cm	56 (54.9)	28 (50.0)	
Serum CEA			
≥ 5 ng/mL	45 (44.1)	26 (57.8)	0.548
< 5 ng/mL	57 (55.9)	29 (50.9)	
Type of surgery			
LAR	77 (75.5)	42 (76.3)	0.215
APR	3 (2.9)	3 (5.5)	
Hartmann	1 (1.0)	1 (1.8)	
ISR	21 (20.6)	9 (16.4)	
Complication of surgery, Clavien-Dindo			
0	84 (82.4)	47 (85.5)	0.494
1	4 (3.9)	1 (1.8)	
2	6 (5.9)	2 (3.6)	
3	7 (6.9)	4 (7.3)	
4	1 (0.9)	1 (1.8)	
ypTNM stage			
IIIa	9 (8.8)	7 (77.8)	0.313
IIIb	65 (63.7)	33 (50.8)	
IIIc	28 (27.5)	15 (53.6)	
ypTNM, T stage			
T2	14 (13.7)	10 (71.4)	0.198
T3	66 (64.7)	36 (54.5)	
T4	22 (21.6)	9 (40.9)	
ypTNM, N stage			
N1	60 (58.8)	33 (55.0)	0.794
N2	42 (41.2)	22 (52.4)	
Histologic type			
Adenocarcinoma	90 (88.2)	48 (53.3)	0.872
Signet ring cell	8 (7.8)	5 (62.5)	
Mucinous	4 (3.9)	2 (50.0)	
Histologic grade			
Well/ Moderate	70 (68.6)	37 (52.9)	0.750
Poorly	32 (31.4)	18 (56.3)	
Number of ELN			
≥ 12	79 (77.5)	42 (53.2)	0.776
< 12	23 (22.5)	13 (56.5)	
Metastatic LNR			
≥ 0.3	40 (39.2)	19 (47.5)	0.296
< 0.3	62 (60.8)	36 (58.1)	
Regimen of Adjuvant Chemotherapy			
5FU/LV	51 (50)	24 (47.1)	0.164
FOLFOX	51 (50)	31 (60.8)	
ECOG (before treatment)			
0	32 (31.4)	17 (53.1)	0.362
1	63 (61.8)	32 (50.8)	
2	6 (5.9)	6 (100)	

*UFUR* tegafur-uracil, *CEA* carcinoembryonic antigen, *LAR* lower anterior resection, *APR* combined abdominal-perineal resection, *ISR* intersphincteric resection, *ELN* total examined nodes number, *LNR* the ratio of metastatic lymph nodes to the total examined nodes number

The Clavien-Dindo Classification: https://www.assessurgery.com/clavien-dindo-classification/

**Table 2:** Continuous variable of patients with or without UFUR maintenance for ypstage III rectal cancer

Variable	with UFUR maintenance Median (Range)	without UFUR maintenance Median (Range)	*P value*
Age	64.7 (35–86)	68.0 (13–83)	0.185
BMI	24.1 (16.3–31.9)	24.2 (13.2–34.0)	0.675
CEA, ng/mL	4.8 (0.5–116.5)	4.0 (0.4–53.13)	0.212
ELN	14 (4–43)	21 (3–51)	0.029
LNR	0.21 (0.03–0.92)	0.20 (0.02–0.84)	0.782
Tumorlocation, cm	6.63 (1–12)	7.42 (3–12)	0.254
Follow-up (month)	64.0 (19.0–124.0)	73.1 (15.4–148.3)	0.207

*UFUR* tegafur-uracil, *BMI* body mass index, *CEA* carcinoembryonic antigen, *ELN* total examined nodes number, *LNR* the ratio of metastatic lymph nodes to the total examined nodes number

A harvest of ELN ≥12 was defined as an adequate quality in treatment of colorectal cancer. When the continuous variable was transformed into a categorical variable, there was no difference between the maintenance and observation groups. Moreover, 41 of the 55 patients in the maintenance group (74.5%) and 37 of the 47 patients in the observation group (78.7%) had adequate ELN (*p* = 0.447). Adequate ELN (≥12) has been considered a critical prognostic factor in surgical treatment for colorectal cancer. As presented in Table 1, the adequate ELN did not differ between the observation and maintenance groups. As presented in Table 2, the median and range of LNR were also similar between the two groups. The potential bias for the decision of tegafur–uracil maintenance and for survival outcome was probably minimized in our database.

### Follow-up and survival

**Fig. 1:**
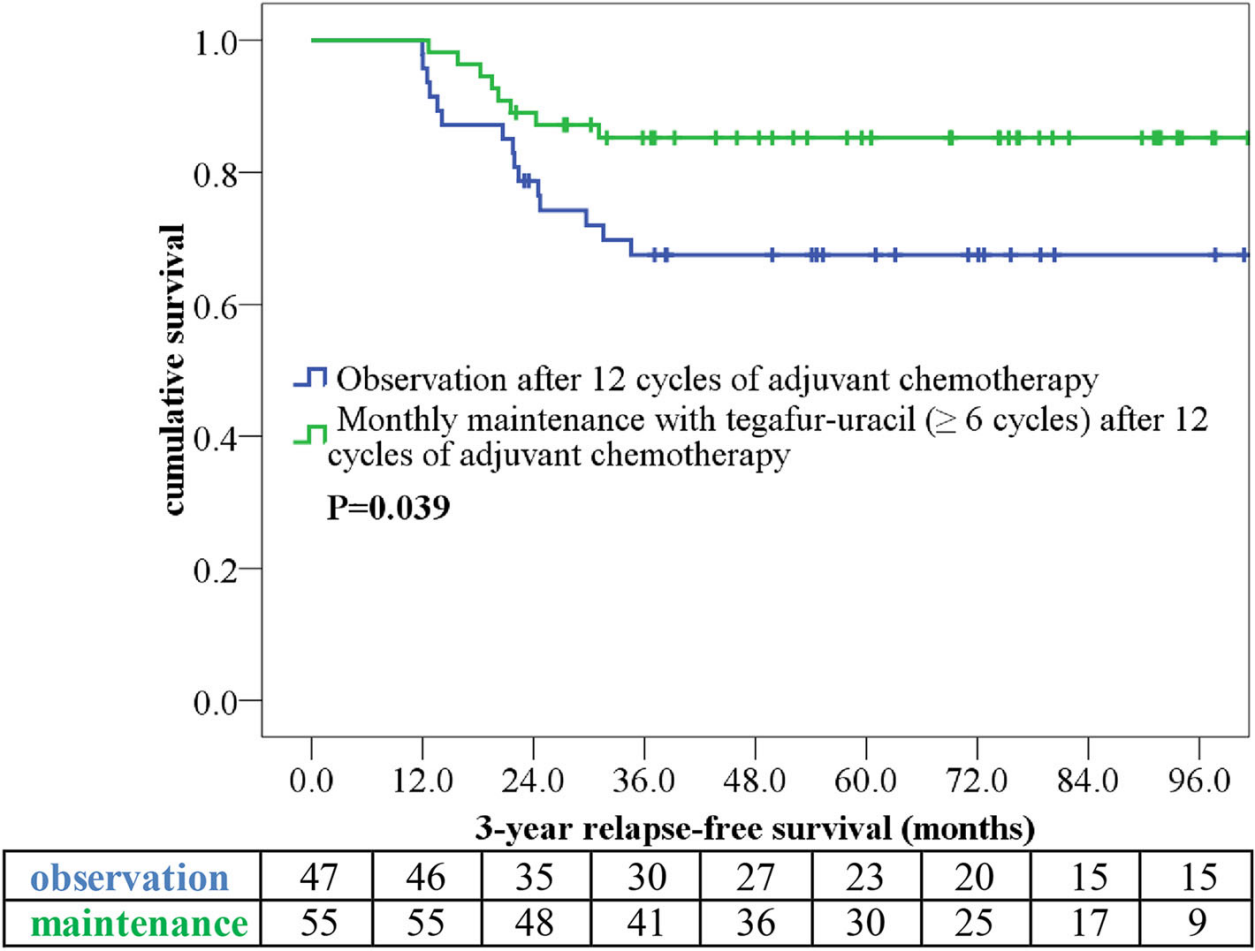
Kaplan–Meier 3-year relapse-free survival curve of ypStage III rectal cancer patients. Patients were stratified according to maintenance therapy with monthly tegafur–uracil (with vs. without)

**Fig. 2:**
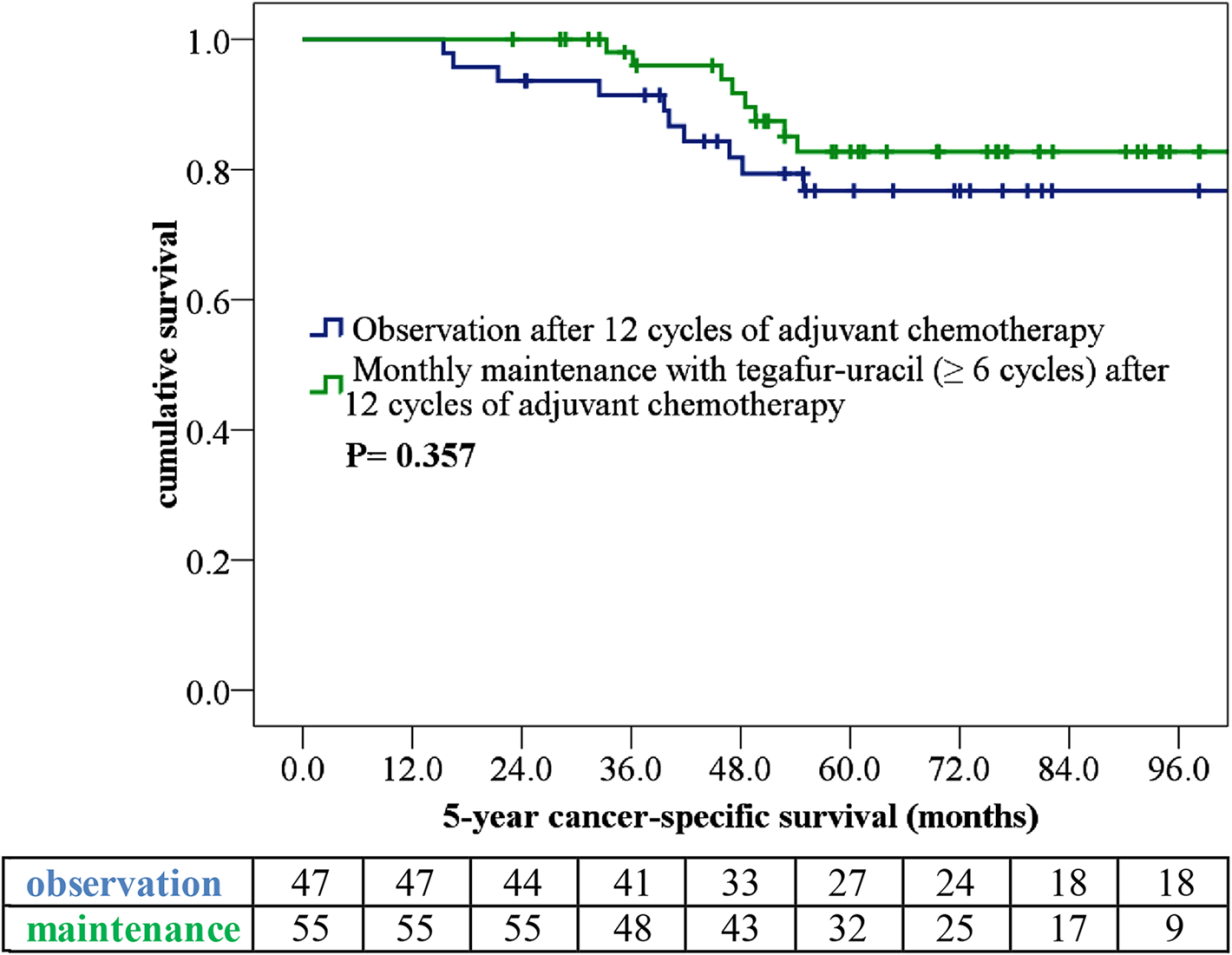
Kaplan–Meier 5-year cancer-specific survival curve of ypStage III rectal cancer patients. Patients were stratified according to maintenance therapy with monthly tegafur–uracil (with vs. without)

The median (range) follow-up durations were 69.7 (15.4–148.3), 64.0 (19.0–124.0), and 73.1 (15.4–148.3) months for all patients, those in the maintenance group, and those in the observation group, respectively. We compared the 3-year RFS and 5-year CSS in patients receiving tegafur–uracil maintenance for ≥6 cycles with those who received observation only after adjuvant chemotherapy. The 3-year RFS observed in the maintenance group (85.1%) was longer than that observed in the observation group (67.5%; *p* = 0.039; Fig. 1). We noted that cancer relapse occurred in 30 patients during follow-up (Table 3), in addition to observing that the observation group had a higher percentage of distant metastasis. Eight patients could undergo metasectomy (lung = 2, liver = 5, and local recurrence = 2). Of 30 patients with cancer relapse, 8 had CSS > 5 years due to effective palliative chemotherapy and metasectomy. The 5-year CSS that observed in the maintenance group (81.9%) was determined to be longer than that noted in the observation group (73.9%; *p* = 0.357; Fig. 2).

**Table 3:** Cancer relapse in UFUR maintenance and observation groups

	With UFUR maintenance (*n* = 55)	Without maintenance (*n* = 47)	
	Patient number (%)	Patient number (%)*P value*	
Relapse-location			
Local	3 (5.5)	3 (6.4)	0.843
Distant	10 (18.2)	16 (34.0)	0.067
Relapse-organ			
Liver	7 (12.7)	8 (17.0)	0.542
Lung	6 (10.9)	10 (21.3)	0.151
Peritoneum	2 (3.6)	2 (4.3)	0.872
Distant lymph nodes	1 (1.8)	1 (2.1)	0.911

**Fig. 3:**
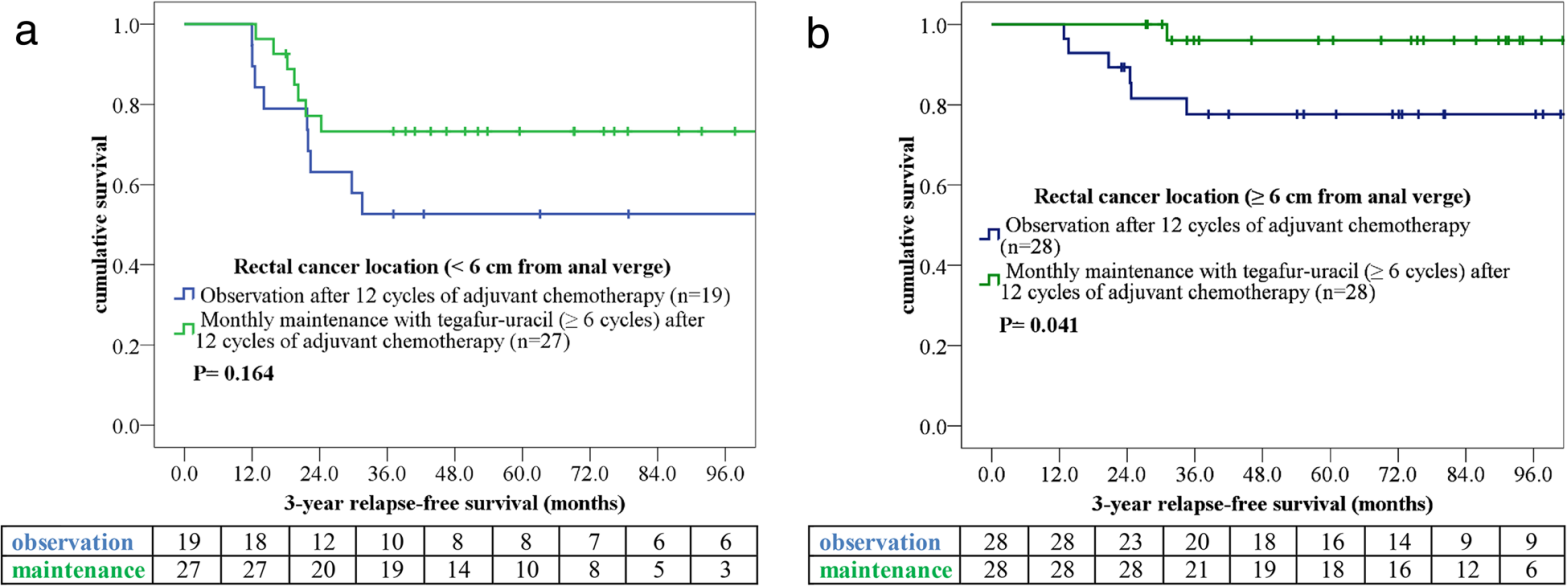
Patients undergoing monthly tegafur–uracil treatment were stratified according to different tumor locations: **a** < 6 and **b** ≥6 cm from the anal verge

**Fig. 4:**
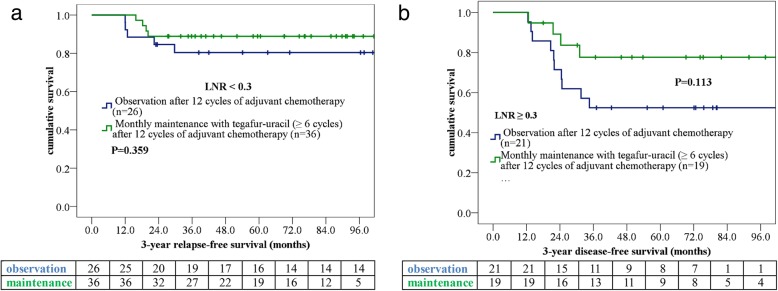
Patients undergoing monthly tegafur–uracil treatment were stratified according to different metastatic lymph node ratios: **a** < 0.3 and **b** ≥0.3

Through a multivariate analysis executed using a forward stepwise Cox regression model, we verified lower rectal cancer location (< 6 cm from the anal verge), higher metastatic lymph node ratio (LNR; ≥0.3), and no maintenance therapy with tegafur–uracil to be independent prognostic factors for a poorer 3-year RFS (Table 4). To investigate the effect of tegafur–uracil maintenance on the RFS associated with different independent factors, we further analyzed tegafur–uracil maintenance in patients with higher LNR or lower rectal cancer location. Higher rectal cancer location (≥6 cm from the anal verge) increased the survival benefit of tegafur–uracil maintenance (*p* = 0.041, Fig. 3). However, the benefit from tegafur–uracil maintenance was only a trend of a longer RFS in patients with LNR ≥ 0.3 (*p* = 0.113, Fig. 4).

**Table 4:** Results of a Cox proportional hazard model to identify the significant variables of disease-free survival

Variable	3-Year Relapse-Free Survival (%)	3-Year Relapse-Free Survival Analysis	
		*P* value	HR (95% CI)
Tumor Location			
(< 6 vs. ≥6 cm)	64.3% vs. 86.9%	0.001	4.944 (1.922–12.721)
LNR			
(≥0.3 vs. < 0.3)	63.1% vs. 85.4%	0.024	2.662 (1.136–6.236)
UFUR maintenance			
With vs. Without	85.1% vs. 67.5%	0.024	0.371 (0.157–0.880)

Age (< 65 vs. ≥65 years), Sex (male vs. female), CEA (≥5 vs. < 5 ng/mL), ypTNM (T and N stage), Histologic Grade (Moderate & Well vs. Poor),BMI (underweight, normal and overweight),Histologic Type (Adenocarcinoma, Mucinous adenocarcinoma and Signet ring cell) and regimen of adjuvant chemotherapy (5FU or FOLFOX) were variables without significant *P*-value in forward stepwise regression

*UFUR* tegafur-uracil, *LNR* the ratio of metastatic lymph nodes to the total examined nodes number

## Discussion

In a study on patients with nonmetastatic rectal cancer [18], fluoropyrimidine-based chemotherapy reduced disease recurrence risk (25%) in patients undergoing adjuvant chemotherapy relative to those subjected to observation alone in a Cochrane database. This result confirms that implementing adjuvant chemotherapy based on 5-FU can increase survival in nonmetastatic rectal cancer patients. However, the precise influence exerted by maintenance chemotherapy on patients diagnosed as having stage III cancer of the rectum remains unclear. Moreover, in the context of colorectal cancer, reports on maintenance therapy with tegafur–uracil executed after adjuvant chemotherapy are scant.

In two retrospective studies on patients diagnosed as having stage III cancer of the colon, the 5-year OS and 3-year DFS were significantly longer in the tegafur–uracil maintenance group than those in the observation group [19, 20]. Furthermore, to our knowledge, only a few studies have evaluated the effect of tegafur–uracil maintenance on survival benefits in patients diagnosed as having ypStage III cancer of the rectum. After monthly tegafur–uracil was added to maintenance therapy in the present study, we found that the 3-year RFS of the ypStage III rectal cancer patients increased significantly. In the forward stepwise Cox regression model, we determined six cycles of monthly tegafur–uracil maintenance therapy to constitute an independent prognostic factor for 3-year RFS.

The survival benefit may result from the distinct inhibitory effect of tegafur–uracil on tumor metastasis. Thus, maintenance therapy may reduce the occult tumor burden, along with reducing the potential for cancer relapse. There are several possible modes of action for maintenance or metronomic therapy in cancer treatment. Metronomic therapy activated innate antitumor immunity, which included the depletion of immune-suppressive regulatory T cells, activation of antitumor CD8^+^ T cells, and maturation of dendritic cells, and then induced tumor regression [21]. Maximum-tolerated-dose chemotherapy could induce higher suppression of natural killer and T-cytotoxic cells compared with low-dose metronomic therapy. After its immunomodulatory action, low-dose metronomic therapy showed a therapeutic benefit that was superior to that of maximum-tolerated-dose chemotherapy in both early and advanced metastatic disease [22].

In addition to the influence on and modulation of cellular immunity, the use of a chemotherapeutic agent at a low dose may have desirable effects in cancer treatment. Angiogenesis—regulated by a proangiogenic–antiangiogenic factor balance—is a crucial process involved in mammalian development [23] and metastatic tumor growth. If there are insufficient angiogenetic factors to supply adequate blood perfusion for occult metastasis, tumor growth is inhibited and apoptosis occurs. Tumors with low-angiogenesis phenotypes, such as lower microvessel density, have been proposed as prognostic factors in various cancers in humans, such as lung [24], gastric [25], breast [26], and colorectal [27] cancer. Tumor angiogenesis may be inhibited by tegafur–uracil administration in vivo [28]. This inhibitory effect on angiogenesis may depend on the signaling pathway–vascular endothelial growth factor (VEGF) cross linkage. Tumor cell–derived angiogenetic factor levels may be persistently inhibited through the anticancer effects of tegafur–uracil or 5-FU [29, 30]. As recently revealed by Kerbel et al., low-dose metronomic chemotherapy sustained the suppression of endothelial progenitor cells and increased the levels of thrombospondin-1, a potent angiogenesis inhibitor [31].

Intravenous adjuvant chemotherapy at the maximum tolerated dose can elicit strong antitumor effects on proliferative cancer cells. The proportion of nonproliferating tumor cells may have a poor response to short-term adjuvant therapy. Long-term tegafur–uracil administration after adjuvant chemotherapy likely inhibits cancer relapse through the antitumor effects associated with antiangiogenesis. In our study of ypStage III rectal cancer, monthly tegafur–uracil administration for ≥6 cycles as a maintenance treatment after adjuvant chemotherapy also resulted in an improved 3-year RFS. This RFS benefit might be supported by the aforementioned mechanisms, but the potential mechanisms underlying the influence of maintenance or metronomic therapy on cancer treatment require elucidation. However, a contradictory result was reported by Tas et al. [32], whose major finding was that maximum-tolerated-dose chemotherapy, but not low-dose metronomic chemotherapy, resulted in significant changes in serum VEGF, thrombospondin-1, and VEGF receptor 1 (VEGFR-1) levels.

Some limitations inherent in this study are outlined as follows: (1) This was a single-institute retrospective study performed using a relatively small database. (2) The distribution of variable factors between the two comparison groups did not differ significantly; however, this distribution was not completely random as required for prospective randomized control trials. (3) The tegafur–uracil treatment duration was variable in the enrolled population, and our sample population size was too small; hence, further analysis of the effects on survival according to different durations of tegafur–uracil use was unfeasible. Nevertheless, six cycles of monthly tegafur–uracil treatment can be considered adequate maintenance duration based on a review of several clinical studies [19, 20].

Finally, relevant prospective randomized controlled trials are required for understanding the effects of and issues related to post–adjuvant chemotherapy maintenance therapy in ypStage III rectal cancer patients further. Moreover, the design of such trials would minimize any ethical debates due to the fact that they would not engender any changes in the current treatment standards for locally advanced rectal cancer.

## Conclusions

The addition of monthly tegafur–uracil for ≥6 cycles after preoperative RT (5 × 5 Gy), radical resection, and 12 cycles of intravenous adjuvant chemotherapy (5-FU based regimen) probably increases the 3-year RFS of ypStage III rectal cancer patients.

## Data Availability

The raw data supporting our findings cannot be shared because the use of raw data was limited from previous IRB permit. If any researcher requests our raw data, please contact us (e-mail: ccchin3477@gmail.com or kuoyihung@cgmh.org.tw).

## Abbreviations

5-FU/LV: 5-Fluorouracil plus leucovorin
AJCC: American Joint Committee on Cancer
CRT: Rradiotherapy (radiation dose: 5040 cGy) with chemotherapy
CSS: Cancer-specific survival
DFS: Disease-free survival
ECOG: Eastern Cooperative Oncology Group
FOLFOX: 5-FU/LV plus oxaliplatin
IRB: Institutional Review Board
OS: Overall survival
RFS: relapse-free survival
RT: Radiotherapy (radiationdose, 2500 cGy)
TME: Total mesorectal excision
TNM: Tumor–node–metastasis
